# Specificity and incremental predictive validity of implicit attitudes: studies of a race-based phenotype

**DOI:** 10.1186/s41235-021-00324-y

**Published:** 2021-09-06

**Authors:** Benedek Kurdi, Timothy J. Carroll, Mahzarin R. Banaji

**Affiliations:** 1 Department of Psychology, Harvard University, Cambridge, MA USA; 2 Department of Psychology, Yale University, New Haven, CT USA; 3 TriNetX, Cambridge, USA

**Keywords:** Correspondence principle, Hair texture phenotype, IAT, Implicit Association Test, Implicit attitudes, Predictive validity

## Abstract

Four studies involving 2552 White American participants were conducted to investigate bias based on the race-based phenotype of hair texture. Specifically, we probed the existence and magnitude of bias in favor of Eurocentric (straight) over Afrocentric (curly) hair and its specificity in predicting responses to a legal decision involving the phenotype. Study 1 revealed an implicit preference, measured by an Implicit Association Test (IAT), favoring Eurocentric over Afrocentric hair texture among White Americans. This effect was not reducible to a Black/White implicit race attitude nor to mere perceptual preference favoring straight over curly hair. In Study 2, the phenotype (hair) IAT significantly and uniquely predicted expressions of support in response to an actual legal case that involved discrimination on the basis of Afrocentric hair texture. Beyond replicating this result, Studies 3 and 4 (the latter preregistered) provided further, and even more stringent, evidence for incremental predictive validity: in both studies, the phenotype IAT was associated with support for a Black plaintiff above and beyond the effects of two parallel explicit scales and, additionally, a race attitude IAT. Overall, these studies support the idea that race bias may be uniquely detected by examining implicit attitudes elicited by group-based phenotypicality, such as hair texture. Moreover, the present results inform theoretical investigations of the correspondence principle in the context of implicit social cognition: they suggest that tailoring IATs to index specific aspects of an attitude object (e.g., by decomposition of phenotypes) can improve prediction of intergroup behavior.

Social evaluation draws upon a multitude of cues, many of them seemingly irrelevant, and even opposed, to the task at hand. Notably, aspects of physical appearance reliably predict judgments about ability and character, even when there is no objective basis for such prediction: men with beards are more likely to be seen as angry (Craig et al., 2019); men and women with lower voice pitch are rated to have more leadership potential (Klofstad et al., 2012); men with male pattern baldness are perceived as less likable and assertive (Cash, 1990); tall women are viewed as more intelligent and ambitious (Chu & Geary, 2005); physically attractive people are judged to be more honest and more competent (Dion et al., 1972); and different configurations of facial features, such as the distance between the eyes or the shape of the mouth, predict ratings of likeability, trustworthiness, competence, and aggressiveness (Todorov, 2017; Willis & Todorov, 2006).

Beyond the laboratory studies mentioned above, the effects of physical features on social perception and judgment have also been demonstrated in naturalistic datasets obtained in consequential real-world settings (for a review, see Todorov et al., 2015). For example, CEOs who look more competent receive higher salaries (Graham et al., 2016); West Point cadets who appear more dominant, on average, achieve a higher rank in the military (Mazur et al., 1984); and competent-looking women tend to have less success in online dating (Olivola et al., 2014). Although such physical features show no relationship with actual ability or character, they are nonetheless used to make inferences about individuals, creating systematic inaccuracies in perception and judgment, with considerable impact.

Seemingly irrelevant physical features can become especially potent drivers of social inequality when judgment and behavior^1^ involve disadvantaged social groups. Specifically, work on racial phenotypicality bias (Maddox, 2004) has demonstrated that possessing Afrocentric features, including darker skin, a wider nose, and fuller lips, has far-reaching consequences in both laboratory studies and real-life contexts. For instance, the more Afrocentric a criminal defendant’s facial features, the greater are ratings of aggression (Blair et al., 2005) and the stronger the prediction of future negative behaviors, which is crucial in decisions about parole (Blair et al., 2002). Moreover, skin tone independently produces variation in judgment: dark-skinned Black targets evoke more negative cultural stereotypes in a free response procedure than light-skinned Black targets (Maddox & Gray, 2002), and priming police officers with crime increases the likelihood of their misremembering a Black target to be more Afrocentric in facial features (Eberhardt et al., 2004).

In addition to such laboratory experiments, correlational work using archival data has also provided evidence for a relationship between more Eurocentric features and better educational attainment (Keith & Herring, 1991) and between Afrocentric features and the probability of receiving a death sentence in criminal cases involving White victims (Eberhardt et al., 2006). Crucially, Blair et al. (2004) found that whereas criminal sentencing decisions did not differ across White and Black targets, more Afrocentric features were associated with harsher sentences within each group. In other words, even White defendants with more Afrocentric features were punished more severely than those with more Eurocentric features. These results suggest that specific physical features, such as skin tone or facial cues, carry independent psychological meaning. That is, a complete configuration of features is not necessary to activate group-based stereotypes and attitudes. Rather, racial category membership and racial phenotypicality can become dissociated from each other and hence independently impact social behavior.

In real-life decisions, as well as in experimental studies designed to be ecologically realistic, it is difficult if not impossible to isolate the effects of unique race-based features on social judgment, given that these features are reliably correlated with each other (Maddox, 2004). As such, beyond a limited number of studies experimentally manipulating skin color (Maddox & Chase, 2004; Maddox & Gray, 2002), work attempting to isolate the contributions of specific Afrocentric features to social judgment is largely absent from the literature. Given the significance of hair in the history and culture of the African American community, and especially African American women, the dearth of social psychological research on this particular dimension of racial phenotypicality bias is particularly surprising.

Historically, White Americans have seen Afrocentric,^2^ curly hair as inferior to Eurocentric, straight or wavy hair, and acts of discrimination specifically on the basis of hair texture have been reported and received attention in the media and the press (Abeyta, 2019; Byrd & Tharps, 2014; Hunter et al., 2009; Tabacco Mar, 2018). The repercussions of such discrimination are visible in several aspects of contemporary American culture: African American students wearing natural hairstyles (that is, hairstyles that have not been artificially modified to look more like straight or wavy Eurocentric hair) often face adverse consequences, including social ostracism, suspensions, and other forms of punishment (Abeyta, 2019; Tabacco Mar, 2018). African American women, in particular, continue to face pressure to straighten their hair; according to recent statistics, over 60 percent of them chemically relax their hair in spite of demonstrated health risks (Byrd & Tharps, 2014). At the same time, there have also been signs of progress, including a burgeoning natural hair movement, books written to teach children the goodness of naturally curly, Afrocentric hair (e.g., Cherry, 2019; Tarpley, 2018), and legal victories in the fight against discrimination based on hair texture, including recent ban on such discrimination first in California (Willon & Díaz, 2019) and ten other states subsequently.

Despite the history of isolating Afrocentric hair texture specifically as a target for racial animus and the reverberations of such history to this day, to our knowledge, the effects of hair texture on consequential social judgments have received little attention in psychological research, with two notable exceptions. Opie and Phillips (2015) showed that Black women with straight hair are viewed as more professional and more likely to succeed by both White American and African American perceivers. In a more recent paper by Koval and Rosette (2020), Black women with chemically straightened hair were rated as more competent and professional and more likely to be recommended for a job interview than Black women with natural hair. This effect was especially pronounced when the job was described as one with a relatively strict dress code (consulting) rather than one with a relatively lenient dress code (advertising).

## The present work

In the present project, we conducted a first empirical investigation of implicit evaluations along a single dimension of physical appearance: Eurocentric (straight or wavy) versus Afrocentric (curly) hair texture. To our knowledge, no published work to date has measured implicit attitudes toward specific phenotypic traits rather than toward racial categories in general (e.g., Black Americans and White Americans). We believe that incorporating implicit judgments into the study of how hair texture influences social perception and behavior stands to benefit both our understanding of racial phenotypicality bias (by examining its automatic nature; Greenwald & Banaji, 1995) and our understanding of implicit social cognition (by testing its power in explaining discrimination on the basis of a race-based phenotype).

Explicit and implicit social cognition are theoretically and empirically distinct: The former uses self-report and the latter indirect indices, such as response latencies in categorizing stimuli, to index mental content. As such, implicit measures may bring to light hitherto unexplored aspects of the bias against Afrocentric hair texture. Moreover, given demonstrations of unique predictive validity of implicit and explicit attitudes over and above each other (Greenwald et al., 2009; Kurdi et al., 2019b), newly adding an implicit measure of bias may improve the prediction and explanation of discrimination in the workplace.

In addition, investigating racial phenotypicality bias using implicit measures may also contribute to the construct validation of implicit social cognition: Most studies seeking to predict behavior from implicit measures rely on a generic group-based attitude (e.g., association of good/bad attributes with Black/White categories). However, such studies seem to violate the so-called correspondence principle according to which criterion behaviors and the attitudes used to predict them should match each other in terms of specificity (Ajzen & Fishbein, 1977). In the context of the present studies, expressions of support for a plaintiff in a legal case alleging discrimination on the basis of hair texture may be better predicted by implicit attitudes toward Afrocentric hair texture than by implicit attitudes toward the group, i.e., African Americans as a social category (see also Irving & Smith, 2020).

Driven by these dual objectives, the studies reported below investigated the presence, magnitude, and correlates of an implicit attitude favoring Afrocentric hair texture over Eurocentric hair texture. In addition, we examined the relationship between specific implicit hair attitudes and more general implicit race attitudes (Studies 1, 3, and 4), the relationship between explicit and implicit attitudes toward Afrocentric hair texture (Studies 2–4), and the unique predictive power of explicit and implicit hair attitudes with regard to a relevant intergroup behavior (Studies 2–4).

## Studies 1A and 1B

Study 1A was designed to provide an initial test of implicit attitudes toward Eurocentric hair texture versus Afrocentric hair texture and to probe the relationship of these phenotype-based attitudes with more general implicit attitudes toward White and Black racial categories. Study 1B, in turn, addressed a potential confound emerging from Study 1A: specifically, even if the hair attitude IAT were to reveal an implicit preference for Eurocentric hair texture over Afrocentric hair texture, this preference may be due to a more general preference for straight over curly hair or even for straight over curved shapes. To eliminate this alternative explanation, in Study 1B a hair IAT similar to the one featured in Study 1A was administered to participants, but this time using racially ambiguous (light-skinned and clearly non-Black) targets. In this and all remaining studies, we focus on White American participants given well-documented pro-White/anti-Black racial biases in this group (e.g., Nosek et al., 2007). Data from Black American participants are meta-analytically summarized and reviewed collapsing across all studies below.^3^

### Method

#### Open science practices

All materials, data files, and analysis scripts are available for download from the Open Science Framework (https://osf.io/xn7az/). We report all data exclusions and all measures in all studies. Study 4 was preregistered (https://aspredicted.org/tn45b.pdf). In Studies 1–3, the sample size was determined a priori without conducting formal power analyses. In Study 4, the preregistered sample size was based on a power analysis conducted on the basis of the effect size obtained in Study 3. No intermittent data analyses were performed in any of the studies.

#### Participants

Participants were American volunteers from the Project Implicit educational website (http://implicit.harvard.edu/). 320 participants were recruited to participate in Study 1A, and 319 participants were recruited to participate in Study 1B. In Study 1A, 14 participants who did not complete the Implicit Association Test (IAT; Greenwald et al., 1998) and 7 participants whose response latencies suggested inattentive responding (Greenwald et al., 2003) were excluded from further analyses, resulting in a final sample size of 299. In Study 1B, 15 participants who did not complete the IAT and 2 participants whose response latencies suggested inattentive responding were excluded from further analyses, resulting in a final sample size of 302.

Given theoretically expected differences across White American and non-White American participants (Nosek et al., 2007; Tajfel, 1982) and because sample sizes were not sufficient to analyze data broken down by non-White racial groups, we focus on analyses involving White American participants (*N* = 218 in Study 1A and *N* = 217 in Study 1B). Analyses involving African American participants are reported in a section below collapsing across all studies. As is usual for online samples, women (73.50%) were more heavily represented in the sample than men (26.50%), younger individuals were more heavily represented than older individuals (mean = 33.44 years, *SD* = 15.25 years), and liberal individuals (47.48%) were more heavily represented than conservative individuals (22.71%). These and other demographic variables are available for reanalysis to interested investigators for this and all remaining studies.

#### Materials and measures

Three Implicit Association Tests (IATs; Greenwald et al., 1998) were created for use in this study and subsequent studies. All materials and measures used in this project, including the stimuli used on each IAT, are included in Supplement 2.

*Black hair attitude IAT* Given the focus of the present studies on measuring implicit social evaluations along a single dimension (Eurocentric hair texture vs. Afrocentric hair texture), a new set of images was developed for this IAT. “Straight hair” and “curly hair” were chosen as category labels with full knowledge that there are several other terms to denote hair texture. We selected these terms to clearly distinguish two hair types without relying on terms such as “nappy”, “kinky”, or “wooly”, which may be unknown to White American participants, seen as derogatory, or ambiguous in meaning.

The categories “straight hair” and “curly hair” were each represented by four drawings of the faces of dark-skinned women. Each unique face was represented in both stimulus sets, with only hair texture (Eurocentric vs. Afrocentric) manipulated, thereby controlling for facial identity (see Table 1). Importantly for the present purposes, in a separate norming study (*N* = 201; Supplementary Study 1 in Supplement 1), the majority of participants categorized each of these faces as Black (median = 88.56%). As such, in line with our intentions, this IAT measured the effects of hair texture specifically in the context of Black female targets. In this and all remaining studies, only female faces were used on the hair attitude IAT because women are the main targets of discrimination on the basis of hair texture (e.g., Hunter et al., 2009). Moreover, on the basis of the correspondence principle (Ajzen & Fishbein, 1977), we reasoned that, in the subsequent studies, a hair attitude IAT using only female faces as stimuli may predict views about a legal case involving discrimination against an African American woman better than a hair attitude IAT using stimuli of multiple genders.

**Table 1:**
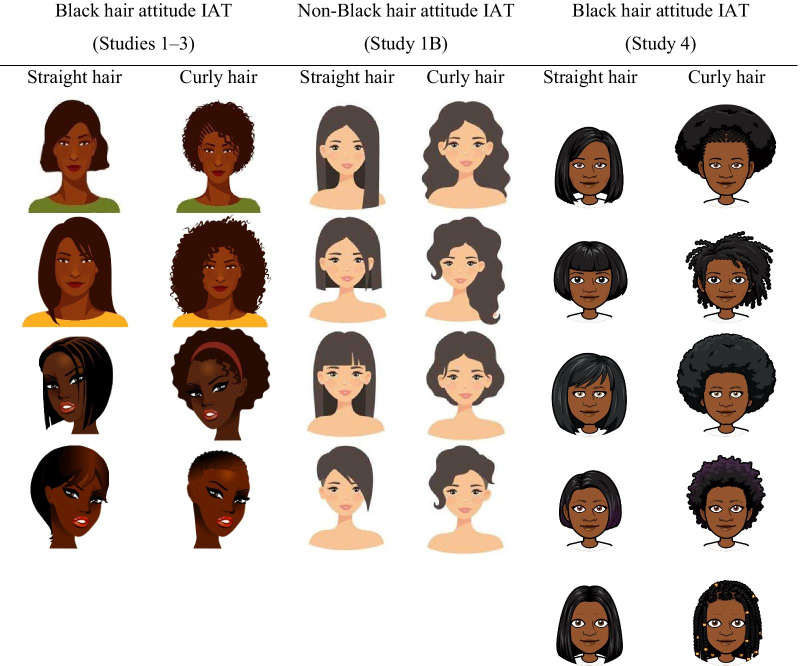
Category stimuli used on the hair attitude IATs in Studies 1–3, Study 1B, and Study 4.

These stimuli were used to measure implicit attitudes toward Eurocentric versus Afrocentric hair texture in a standard 7-block Implicit Association Test (IAT; Greenwald et al., 1998). The order of critical blocks was counterbalanced. The IAT was scored using the improved scoring algorithm (Greenwald et al., 2003), with higher D scores representing higher levels of preference in favor of Eurocentric over Afrocentric hair texture. This IAT showed adequate split-half reliability using even versus odd trials (*r* = . 70).

*Non-Black hair attitude IAT* To probe whether the Black hair attitude IAT described above captures *(a)* a specific evaluation of Eurocentric hair texture versus Afrocentric hair texture, *(b)* a more general evaluation of straight versus curly hair, or *(c)* perhaps even a relatively low-level perceptual preference for straight over curved shapes, an alternative version of the hair attitude IAT was created. Specifically, instead of the line drawings of dark-skinned women, this IAT used line drawings of light-skinned women as target stimuli, varying their hair type between straight and curly (see Table 1). In the same norming study mentioned above (Supplementary Study 1 in Supplement 1), the majority of participants categorized each of these faces as White (median = 55.97%) or Asian (median = 19.41%). Crucially from the perspective of discriminant validity, participants almost never categorized these stimuli as Black (median = 0%). Otherwise, the non-Black hair attitude IAT was identical to the Black hair attitude IAT described above. This IAT showed adequate split-half reliability (*r* = 0.64).

*White/Black race attitude IAT* To further probe the relationship between implicit evaluations of Eurocentric hair texture versus Afrocentric hair texture and implicit evaluations of White and Black racial categories more generally, a standard White/Black race attitude IAT was also created. The two categories were represented using black-and-white photographs of the internal facial features of six individuals from each group (three male and three female faces each; see Table 2). This IAT showed adequate split-half reliability (*r* = 0.66).

**Table 2:**
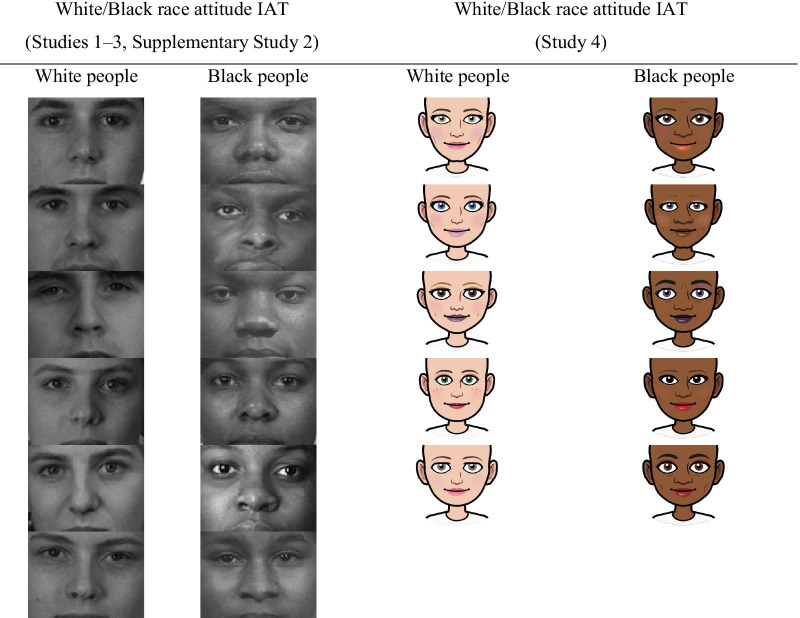
Category stimuli used on the White/Black race attitude IATs in Studies 1–3 and Supplementary Study 2, and Study 4.

#### Procedure

For ease of comparability, the procedure of each study is summarized in Table 3. In Study 1A, participants completed the Black hair attitude IAT and the White/Black race attitude IAT in counterbalanced order. In Study 1B, participants completed the non-Black hair attitude IAT.

**Table 3:** Procedure of Studies 1–4

Study			
Study 1A	Study 1B	Study 2	Studies 3–4
Black hair attitude IAT^*^		Black hair attitude IAT^*^	Black hair attitude IAT^*^
White/Black race attitude IAT^*^			White/Black race attitude IAT^*^
	Non-Black hair attitude IAT		
		Legal case and legal judgments^*^	Legal case and legal judgments^**^
		Self and societal judgments	Self and societal judgments

Each unique measure is presented in a separate row, with the order from top to bottom representing the order of the measures in each study. The order of the measures with the same number of asterisks within each study was counterbalanced. Specifically, In Studies 3 and 4, the Black hair IAT attitude IAT and the White/black race attitude IAT were always administered after each other in counterbalanced order. The legal case and legal judgments were administered either before or after both IATs

### Results

Data from these and all remaining studies are openly available for reanalysis from the Open Science Framework (OSF). Additional results for this and all remaining studies are reported in Supplement 1.

#### Study 1A

*Mean levels* Descriptive statistics for all variables from Study 1A and all remaining studies are provided in Table 4. The mean D score for the Black hair attitude IAT significantly differed from zero, revealing, for the first time, an implicit preference in favor of Eurocentric hair texture over Afrocentric hair texture among White Americans, *t*(217) = 10.50, *p* < 0.001, BF_10_ = 1.10 × 10^18^, Cohen’s *d* = 0.71. Moreover, in line with well-established findings of implicit ingroup preference among White Americans (Nosek et al., 2007), the race IAT revealed significant pro-White attitudes, *t*(217) = 15.82, *p* < 0.001, BF_10_ = 4.95 × 10^34^, Cohen’s *d* = 1.07.

**Table 4:** Means and standard deviations for all variables from Studies 1–4 (White American participants)

	Study 1A		Study 1B		Study 2		Study 3		Study 4	
	Mean	SD	Mean	SD	Mean	SD	Mean	SD	Mean	SD
Black hair attitude IAT	0.30	0.43	–	–	0.32	0.48	0.38	0.52	0.32	0.47
White/Black race IAT	0.39	0.37	–	–	–	–	0.40	0.50	0.34	0.44
Non-Black hair attitude IAT	–	–	0.18	0.58	–	–	–	–	–	–
Self trait judgments	–	–	–	–	4.04	0.62	3.94	0.64	− 7.88	14.75
Societal trait judgments	–	–	–	–	4.99	0.81	5.01	0.87	14.81	15.17
Legal judgments	–	–	–	–	2.60	1.45	2.43	1.43	− 30.00	22.53

Implicit Association Tests (IATs), self trait judgments, and legal judgments were scored such that higher scores indicate higher levels of race bias against Afrocentric hair texture and/or African American targets. Societal trait judgments were scored such that higher scores indicate higher levels of awareness of societal bias. The theoretical range of IAT scores was [− 2; 2], whereas the theoretical range of the explicit judgments was [1; 7] in Studies 1–3 and [− 50; 50] in Study 4

*Relationship between the hair attitude and race attitude IATs* Next, we probed whether implicit hair attitudes and implicit race attitudes are completely independent of each other, related to each other but not redundant, or fully redundant. Scores on the hair and race attitude IATs were found to be significantly correlated, *r* = 0.28 [0.16; 40], *t*(216) = 4.32, *p* < 0.001, BF_10_ = 9.61 × 10^2^, thus providing evidence against the idea of complete independence. Those who showed greater preference for straight hair relative to curly hair also showed preference for White Americans relative to Black Americans.

However, given attenuation as a result of imperfect reliability (Spearman, 1904), bivariate correlations cannot provide conclusive evidence about whether two measures are merely related to each other or redundant. Therefore, we partitioned the variance in implicit hair attitudes into error variance, variance explained by implicit race attitudes, and meaningful unexplained variance (Kurdi et al., 2019a). Although implicit race attitudes accounted for a significant portion of the variance in implicit hair attitudes, *p* = 0.15 [0.04; 0.33], a large portion of meaningful variance remained unexplained, *p* = 0.61 [0.41; 0.73]. As such, these analyses reveal a significant association, but no redundancy, between implicit race and hair attitudes.

#### Study 1B

The non-Black hair attitude IAT revealed an implicit preference for straight over curly hair, *t*(216) = 4.64, *p* < 0.001, BF_10_ = 1.92 × 10^3^, Cohen’s *d* = 0.32. This result suggests that at least some of the preference for straight over curly hair obtained in Study 1A may not have been specific to Black targets. However, if the effect were fully reducible to a decontextualized aesthetic preference, responding on the non-Black hair IAT (Study 1B) and on the Black hair IAT (Study 1A) should have been indistinguishable from each other. Contrary to this prediction, implicit preferences on the non-Black hair IAT were significantly less strong than implicit preferences on the Black hair IAT, *t*(395.44) = − 2.44, *p* = 0.015, BF_10_ = 1.86, Cohen’s *d* = 0.23. As such, the two studies combined suggest that, to a considerable degree, the IAT administered in Study 1A captured implicit preferences specifically in favor of Eurocentric over Afrocentric hair texture.

### Discussion

Study 1A established an implicit preference of considerable magnitude for Eurocentric hair texture over Afrocentric hair texture among White American participants. This preference was moderately related to, but not redundant with, more general implicit evaluations of the underlying racial categories. The results of Study 1B, in turn, suggest that the phenotype-based implicit attitudes observed in Study 1A cannot be fully reduced to an aesthetic preference for straight over curly hair or, even more generally, straight over curved shapes given that the bias was significantly attenuated when the targets were light-skinned racially ambiguous (but clearly non-Black) women rather than dark-skinned Black women. Whether the difference in implicit attitudes between the two studies emerged due to skin tone, facial features, the combination of both, or some other reason, can be more specifically explored in future work. From our perspective, the important finding is that of discriminant validity, i.e., a markedly stronger implicit preference for straight over curly hair texture in the context of Black, rather than non-Black, targets.

Interestingly, the preference for straight over curly hair was not eliminated even when racially ambiguous light-skinned non-Black women, rather than Black women, served as targets on the IAT. This result echoes findings obtained using explicit measures in recent work (Koval & Rosette, 2020): in these studies, both light-skinned White women and dark-skinned Black women tended to be evaluated more negatively when presented with curly, rather than straight, hair; however, similar to the present work involving implicit measures, the effect was considerably more pronounced for Black than White targets. As such, the remaining studies examined the nature of this phenotype-based implicit bias specifically in the context of African American women.

## Study 2

Study 1 demonstrated the existence and magnitude of an implicit preference for Eurocentric hair texture over Afrocentric hair texture among White Americans. Moreover, it provided evidence that this implicit preference cannot be reduced to implicit racial preferences or perceptual or aesthetic preferences for straight over curly hair. Study 2 was designed to further investigate the nature of implicit hair attitudes by probing their relationship with explicit judgments regarding Afrocentric hair texture and White American participants’ views about a specific legal case involving an African American plaintiff who was fired for refusing to alter her natural hair.

Specifically, in the focal analysis of this study, we sought to determine whether implicit hair attitudes can uniquely predict expressions of support for the plaintiff in the legal case over and above two scales of explicit judgments. One of these scales measured explicit trait judgments of women along the dimensions of attractiveness, caring, competence, and professionalism. Based on ubiquitous findings of explicit attitudes predicting intergroup behaviors (e.g., Talaska et al., 2008), we expected this scale to relate to expressions of support in the legal case. In addition, we included a scale measuring participants’ awareness of societal discrimination against Afrocentric hair textures. Such awareness may be expected to be negatively associated with biased behavior (e.g., Pope et al., 2018). Notably, expressions of support provide a conservative test of incremental predictive validity of the Black hair IAT over and above parallel explicit measures: the criterion behavior is procedurally similar to the explicit judgments and, unlike the IAT used to index implicit evaluations, highly controllable.

### Method

#### Participants

The participants were 919 American volunteers recruited from Project Implicit (http://implicit.harvard.edu/). 43 participants who did not complete the IAT and 12 participants whose response latencies suggested inattentive responding were excluded from further analyses, resulting in a final sample size of 864. The analyses reported below focus on 622 White American participants. Similar to Study 1, these participants tended to be female (68.08%) rather than male (31.92%), younger rather than older (mean = 38.87 years, *SD* = 15.42 years), and liberal (53.54%) rather than conservative (19.14%).

#### Materials and measures

In addition to the Black hair IAT already used in Study 1A (split-half reliability: *r* = 0.73), some new materials and measures were created for use in this study, including the description of a legal case involving an African American plaintiff alleging discrimination on the basis of hair texture, and three explicit scales measuring self trait judgments and societal trait judgments about women with Afrocentric hair texture, as well as participants’ views about the legal case mentioned above. All materials and measures used in this study, including the verbatim text of the legal case, are included in Supplement 2.

*Description of legal case* A vignette based on an actual legal case (*Equal Employment Opportunity Commission* v. *Catastrophe Management Solutions*, 2016) was created. The case involved Chastity Jones, an African American woman, who was about to start working at a company in Alabama. However, after she had accepted the position, the corporation informed her that she would need to cut her dreadlocks to comply with company policy. When Jones refused to do so, the company rescinded its offer of employment. Jones sued the company for discrimination. The case was presented to participants without describing the court’s decision, which was to rule in favor of the corporation.

The description of the case provided excerpts from the briefs on behalf of the employer and the plaintiff. Specifically, the employer argued that although “Title VII prohibits discrimination on the basis of immutable (unchangeable) characteristics, such as race, color, or natural origin”,… “[a] hairstyle, even one more closely associated with a particular ethnic group, is a mutable (changeable) characteristic.” In addition, they referred to their corporate policy prohibiting “excessive hairstyles or unusual colors.” The plaintiff, in turn, argued that dreadlocks were a natural part of the bodies of many people of color. As such, “… discrimination against natural Black hair is equivalent to discrimination against skin color.”

### Explicit judgments

*Self trait judgments* A four-item scale was created to measure participants’ personal trait judgments of women with Afrocentric hair texture in terms of how attractive, caring, competent, and professional they are. For each of these traits, participants were presented with the incomplete sentence “I BELIEVE that dreadlocks and afros make women look…” and selected one of seven response options. For instance, for the attractiveness trait, the response options read “much less attractive”, “moderately less attractive”, “slightly less attractive”, “no different in terms of attractiveness”, “slightly more attractive”, “moderately more attractive”, and “much more attractive.” For the remaining traits, the word “attractive” was replaced with the corresponding trait adjective. Responses were scored from 1 to 7 such that higher scores correspond to higher levels of explicit bias against Afrocentric hair texture. The self trait judgment scale exhibited satisfactory internal consistency (Cronbach’s α = 0.75).

*Societal trait judgments *A four-item scale was created to measure participants’ perceptions of general societal views of women with Afrocentric hair texture. For each of the four traits mentioned above, participants were presented with the incomplete sentence “SOCIETY BELIEVES that dreadlocks and afros make women look…” and selected one of seven response options, as described above. Responses were scored from 1 to 7 such that higher scores correspond to higher levels of perceived societal bias against Afrocentric hair texture. The societal trait judgment scale exhibited satisfactory internal consistency (Cronbach’s α = 0.78).

*Views about the legal case* A five-item scale was created to measure participants’ views regarding the legal case described above. The items measured participants’ agreement with (*a*) the plaintiff being correct for suing the company, (*b*) the plaintiff showing disregard for the company, (*c*) the company showing race bias against the plaintiff, (*d*) discrimination based on hair being equivalent to racial discrimination, and (*e*) the company being within its rights to not hire the plaintiff based on her hairstyle. Participants rated their agreement or disagreement using a 7-point Likert scale, with the response options anchored by “strongly disagree” and “strongly agree.” Responses were scored such that higher scores indicated higher levels of agreement with the company, i.e., items (*a*), (*c*), and (*d*) were reverse scored. The legal judgment scale exhibited satisfactory internal consistency (Cronbach’s α = 0.85).

#### Procedure

Participants completed the Black hair attitude IAT, read the legal case, and completed the legal judgment scale. The legal judgment scale always directly followed the legal case, and the order of the IAT and the legal case and judgments was counterbalanced. Finally, participants completed the self and societal judgment scales. The two scales were interleaved with each other such that items for self and society for the same trait always followed each other in individually randomized order.

#### Analytic strategy

Given satisfactory internal consistency of each scale, the analyses involving mean levels were performed on composite variables. In line with established practice, the structural equation model (SEM) included each item separately as an indicator of a latent variable. For the IAT, two indicator variables were created by calculating D scores using odd-numbered and even-numbered trials, respectively. The correlation matrix forming the basis of the SEM analysis is reported in Supplementary Table 2 (Supplement 1).

### Results

#### Mean levels

The hair attitude IAT revealed significant implicit preference in favor of Eurocentric over Afrocentric hair texture, *t*(614) = 16.51, *p* < 0.001, BF_10_ = 3.01 × 10^47^, Cohen’s *d* = 0.67, replicating the result obtained in Study 1A.

The contrast to explicit judgments is noteworthy: Unlike mean implicit attitudes, mean self trait judgments did not deviate from neutrality, *t*(598) = 1.60, *p* = 0.109, BF_01_ = 6.06, Cohen’s *d* = 0.07. That is, participants reported that Afrocentric hair texture did not influence their social judgments of women in a positive or negative direction. By contrast, mean societal trait judgments were significantly higher than the midpoint of the scale, *t*(598) = 29.97, *p* < 0.001, BF_10_ = 2.13 × 10^177^, Cohen’s *d* = 1.22, indicating awareness of societal bias against Afrocentric hair texture. The mean level of perceived societal bias significantly exceeded the mean level of bias reported for the self, *t*(598) = 24.31, *p* < 0.001, BF_10_ = 3.70 × 10^87^, Cohen’s *d* = 0.99. This finding is reminiscent of the bias blind spot, i.e., the tendency to perceive no bias in the self along with reports of high levels of bias in others (Pronin et al., 2004). We note that mean performance on the hair attitude IAT sits squarely between self-report of own bias and self-report of societal bias.

Finally, mean legal judgments were significantly below the midpoint of the scale, *t*(598) = − 23.63, *p* < 0.001, BF_10_ = 7.28 × 10^83^, Cohen’s *d* = 0.97, indicating general agreement with the African American plaintiff rather than the employer.

#### Prediction of legal judgments

**Fig. 1:**
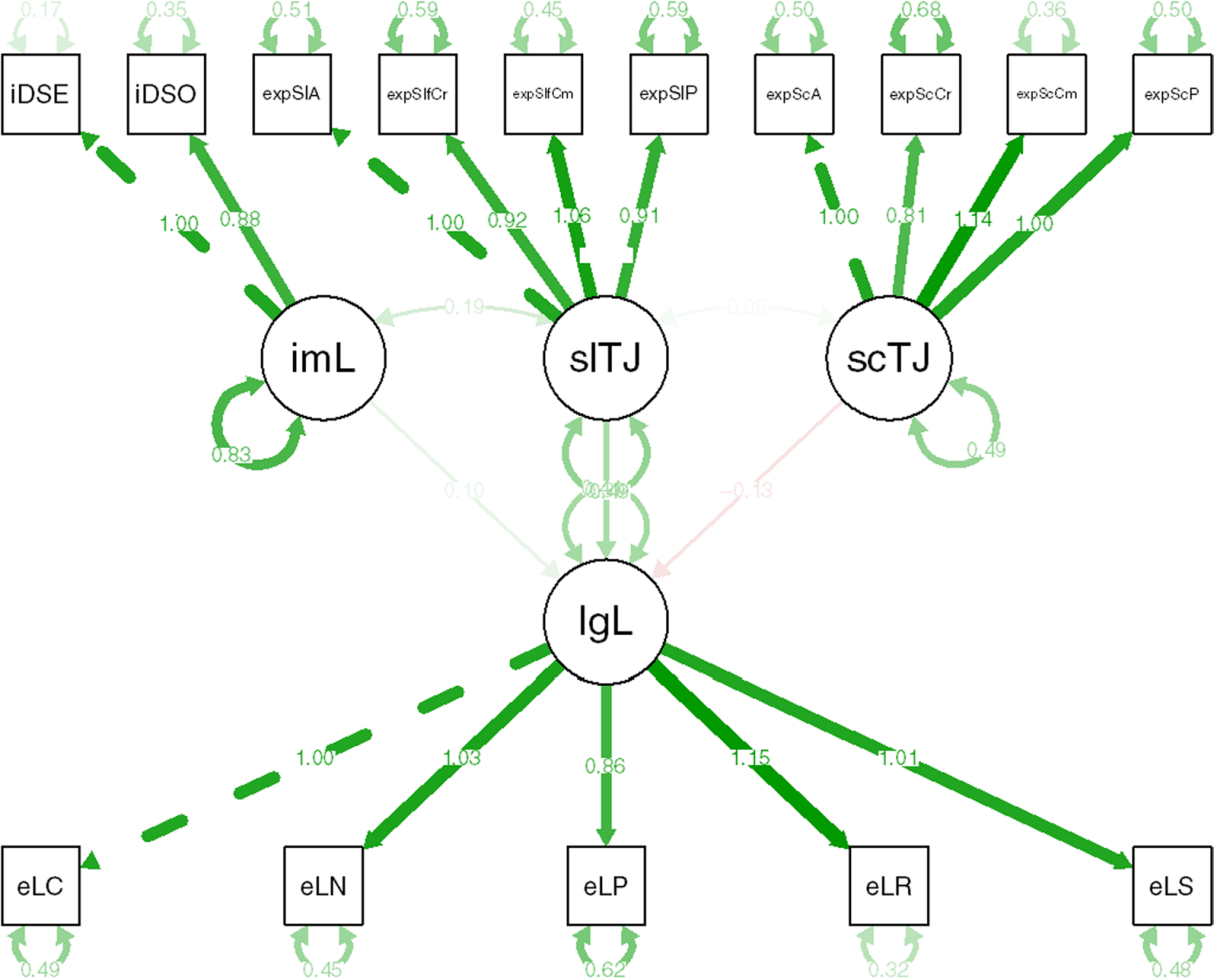
Structural equation model (Study 2) in which a latent legal judgment variable [lgL] is predicted by a latent implicit hair attitude variable [imL], a latent self trait judgment variable [slTJ], and a latent societal trait judgment variable [scTJ]

In line with recent recommendations (Kurdi et al., 2019b; Westfall & Yarkoni, 2016), we probed incremental predictive validity of each measure using a structural equation modeling (SEM) approach.^4^ In the SEM, a latent legal judgment variable was predicted by a latent self trait judgment variable, a latent societal trait judgment variable, and a latent implicit hair attitude variable (see Fig. 1). The model-implied correlation matrix was found to significantly differ from the empirical correlation matrix, *χ*^*2*^(84) = 285.93, *p* < 0.001. However, given inherent conceptual difficulties in interpreting a nonsignificant test as an indication of good model fit (Brown, 2015; Kline, 2010), we preferentially rely on alternative measures. These alternative measures uniformly indicated good, or at least acceptable, fit (see Supplementary Table 3). As such, we proceed with model interpretation.

In line with expectations and given the high degree of both conceptual and procedural overlap, legal judgments were found to be uniquely predicted by both self trait judgments, *β* = 0.39 [0.28; 0.51], *z* = 6.61, *p* < 0.001, and societal trait judgments, *β* = − 0.13 [− 0.22; − 0.03], *z* = − 2.51, *p* = 0.012. Specifically, those with higher levels of self-reported preference for Eurocentric hair texture over Afrocentric hair texture were more likely to side with the corporation in the legal case, whereas those with higher levels of acknowledgement of societal bias were more likely to side with the plaintiff. Remarkably, in spite of no overlapping method variance, the hair IAT significantly and uniquely predicted legal judgments, *β* = 0.10 [0.02; 0.18], *z* = 2.50, *p* = 0.012. Specifically, those with more negative implicit attitudes toward Afrocentric hair texture were less likely to support the plaintiff in her case against the corporation.

### Discussion

Study 2 provides new insights into the nature of phenotype-based implicit attitudes by exploring their relationship with phenotype-based explicit judgments and a relevant measure of behavior among White American participants. In terms of mean levels, the data from Study 2 revealed a dissociation between the explicit and implicit measures of attitude: Whereas the former exhibited no preference, the latter showed relative positivity toward Eurocentric hair texture and relative negativity toward Afrocentric hair texture. This pattern of results is common in the implicit social cognition literature where implicit measures can reveal deviations from neutrality even in the absence of any explicit bias (Nosek et al., 2007).

In addition, Study 2 also suggests that phenotype-based implicit attitudes can be associated with relevant measures of behavior. Specifically, in this study, implicit hair attitudes predicted expressions of support toward a Black plaintiff who sued a corporation because she was required to alter her hairstyle to retain an offer of employment. The fact that implicit hair attitudes were significantly and uniquely related to such expressions of support is remarkable given that, unlike the explicit scales of self and societal trait judgments, the IAT shares little if any procedural overlap with the criterion measure.

## Study 3

Study 2 demonstrated a surprising and unique association of implicit hair attitudes, above and beyond explicit self and societal trait judgments, with expressions of support toward a Black plaintiff in a relevant legal case. In Study 3, we probed whether this association would be sufficiently robust to be replicated in a different sample of White American participants. Moreover, Study 3 also newly included a standard race IAT as a control. In line with the correspondence principle established in the study of explicit attitudes (Ajzen & Fishbein, 1977), we expected that behavior relating to Afrocentric hair texture might be better predicted by implicit attitudes specifically toward Afrocentric hair texture rather than more generally toward African Americans as a category.

### Method

The participants were 753 American volunteers recruited from Project Implicit (http://implicit.harvard.edu/). 34 participants who did not complete the IAT and 9 participants whose response latencies suggested inattentive responding were excluded from further analyses, resulting in a final sample size of 710. The analyses reported below focus on 507 White American participants. Similar to Studies 1–2, these participants tended to be female (65.22%) rather than male (34.78%), younger rather than older (mean = 38.60 years, *SD* = 14.22 years), and liberal (57.99%) rather than conservative (17.36%).

The procedure was similar to that of Study 2, with one exception: in addition to the Black hair attitude IAT, participants also completed a White/Black race attitude IAT (see Study 1A), with the order of both IATs counterbalanced. To prevent participant fatigue, the number of IAT blocks was reduced from seven to five (for details, see Supplement 1).

The Black hair attitude IAT (*r* = 0.59) and the White/Black race attitude IAT (*r* = 0.55) showed adequate split-half reliability, although internal consistency was attenuated relative to Studies 1 and 2, presumably due to the smaller number of trials. The self trait judgment scale (Cronbach’s α = 0.78), the societal trait judgment scale (Cronbach’s α = 0.80), and the legal judgment scale (Cronbach’s α = 0.85) exhibited good-to-excellent internal consistency. The correlation matrix forming the basis of the SEM analysis is reported in Supplementary Table 4 (Supplement 1).

### Results

#### Mean levels

The results involving mean levels of all variables were in line with the results obtained in Studies 1–2 and are thus not discussed further.

#### Prediction of legal judgments

Similar to Study 2, a latent legal judgment variable was predicted by a latent self trait judgment variable, a latent societal trait judgment variable, and a latent implicit hair attitude variable. Moreover, a latent implicit race attitude variable was newly added. Despite a significant difference between the model-implied and empirical correlation matrices, *χ*^*2*^(109) = 257.40, *p* < 0.001, all alternative measures of model fit indicated good fit (see Supplementary Table 3). Therefore, we proceed with model interpretation.^5^

**Fig. 2:**
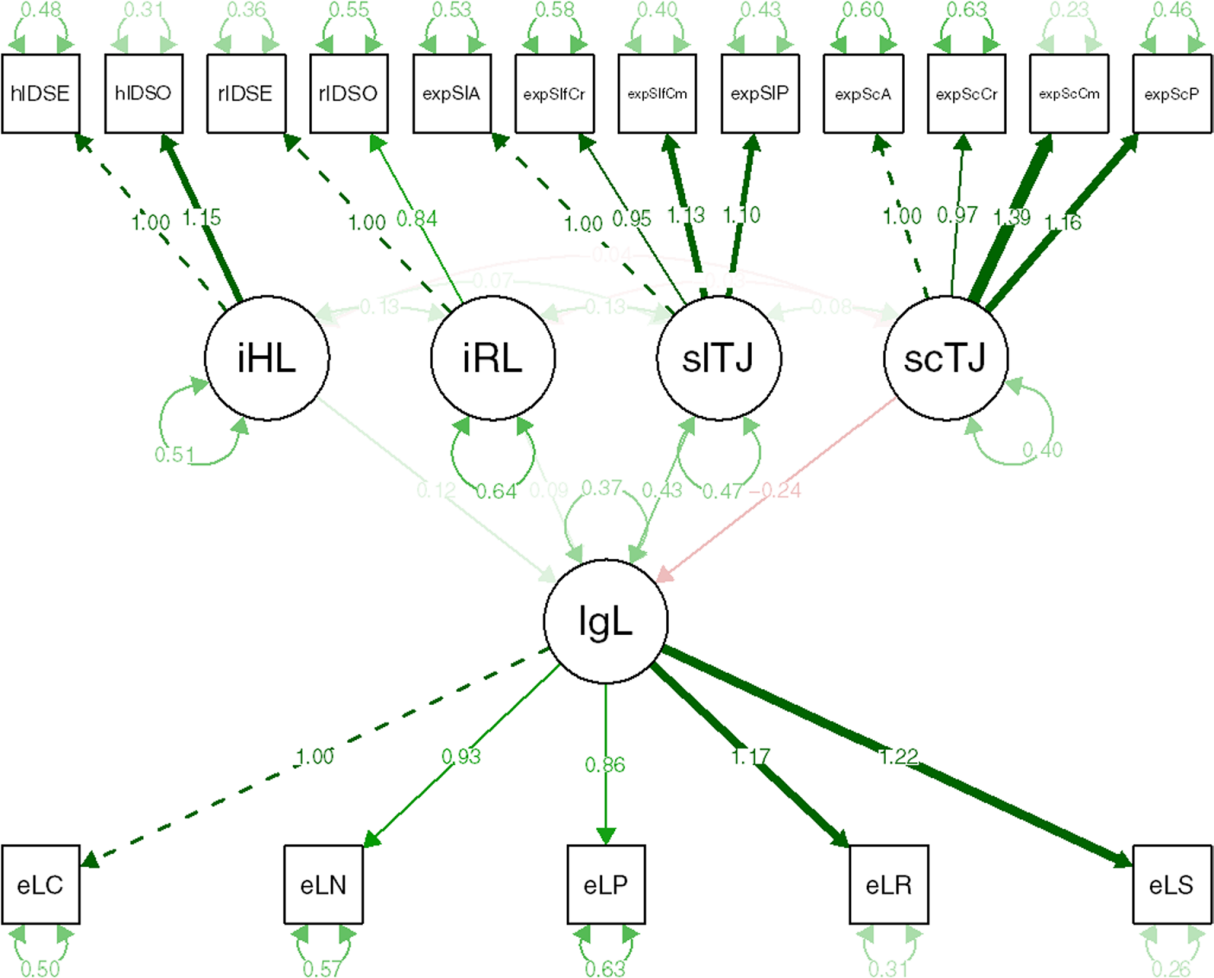
Structural equation model (Study 3) in which a latent legal judgment variable [lgL] is predicted by a latent implicit hair attitude variable [iHL], a latent implicit race attitude variable [iRL], a latent self trait judgment variable [slTJ], and a latent societal trait judgment variable [scTJ]

With regard to self trait judgments, societal trait judgments, and implicit hair attitudes, the results of the SEM model (Fig. 2) replicated the findings of Study 2. Specifically, legal judgments were found to be uniquely and positively predicted by self trait judgments, *β* = 0.43 [0.31; 0.56], *z* = 6.74, *p* < 0.001, and uniquely and negatively predicted by societal trait judgments, *β* = − 0.25 [− 0.26; − 0.13], *z* = − 4.06, *p* < 0.001. That is, the higher their level of self-reported preference for Eurocentric over Afrocentric hair texture and the lower their level of acknowledgement of societal bias, the more likely participants were to agree with the corporation rather than with the plaintiff.

Remarkably, in spite of no shared method variance, implicit hair attitudes were again found to be significantly and uniquely associated with legal judgments, *β* = 0.12 [0.01; 0.22], *z* = 2.13, *p* = 0.033. However, the effect of implicit race attitudes on legal judgments was not statistically significant, *β* = 0.09 [− 0.01; 0.20], *z* = 1.76, *p* = 0.078, suggesting superior predictive validity of the implicit measure specifically tailored to the behavior to be predicted. At the same time, we note that the effect size associated with the two tests is highly similar and, as such, this result should be interpreted with caution, especially in the absence of a formal power analysis.^6^ A more systematic comparison of the effect sizes associated with the hair attitude IAT and the race attitude IAT has been undertaken in an internal meta-analysis reported below.

### Discussion

Study 3 provides an independent replication of the surprising result obtained in Study 2: An implicit measure of phenotypicality bias administered to White American participants significantly and uniquely predicted expressions of support toward an African American plaintiff in a relevant legal case. Notably, this result emerged in the absence of any procedural overlap between the two measures. In addition, the results of Study 3 also provide some indication that the correspondence principle may be operational not only in explicit but also implicit social cognition (see also Irving & Smith, 2020). Specifically, whereas scores on the Black hair IAT were found to be predictive of the criterion behavior, a White/Black race IAT measuring implicit evaluation of the general racial categories was not.

## Study 4

Given the novelty of the approach of applying implicit measures to the study of racial phenotypicality bias, Studies 1–3 should be seen as exploratory. In line with recent recommendations (e.g., Nosek et al., 2018, 2019), in Study 4 we sought to increase the credibility of the main findings emerging from these initial studies by implementing the additional safeguards of a simulation-based power analysis and formal preregistration. In addition, we undertook some other changes to the design of the study to improve internal validity. In Study 3 we compared two IATs that were not matched in the type of face stimuli used. Specifically, grayscale images were used on the race attitude test, whereas line drawings were used on the hair attitude test. Additionally, the race attitude test used faces of both men and women to represent the group, whereas the hair attitude test used only faces of women. To make the two IATs more directly comparable with each other, in Study 4, both tests used line drawings of only female targets as category stimuli. Moreover, we improved the wording of the items used on the two explicit trait judgment scales.

### Method

The participants were 1607 American volunteers recruited from Project Implicit (http://implicit.harvard.edu/). 81 participants who did not complete the IAT and 17 participants whose response latencies suggested inattentive responding were excluded from further analyses, resulting in a final sample size of 1509. In line with the preregistered analysis plan, the analyses reported below focus on 978 White American participants. This target sample size was determined based on a resampling-based simulation approach. Specifically, we aimed to have at least 0.80 power to detect the incremental predictive validity effect associated with the hair attitude IAT in the SEM analysis of Study 3 (*β* = 0.12). Similar to Studies 1–3, participants tended to be female (67.79%) rather than male (30.36%), younger rather than older (mean = 40.80 years, *SD* = 15.28 years), and liberal (52.69%) rather than conservative (25.03%).

The procedure of Study 4 was identical to the procedure of Study 3. However, beyond the formal preregistration and power calculation, some additional changes were implemented to the materials used. Specifically, in Studies 3 and 4, the implicit hair attitude IAT and the implicit race attitude IAT differed from each other in theoretically irrelevant ways, thus creating some potential confounds. Specifically, the hair attitude IAT used line drawings of Black women, whereas the race attitude IAT used black-and-white photographs of the internal facial features of White and Black women and men. To eliminate these unnecessary differences, in Study 4 we used line drawings of women on both tests (see Tables 1 and 2). In addition, the stimuli used for the “curly hair” category on the hair attitude IAT featured line drawings of Black women with curlier hair textures and more stereotypically Black hairstyles than the hair attitude IATs used in Studies 1–3 (see Table 1). Participants responded to all explicit items on 100-point sliding scales rather than 7-point Likert scales and the items on the self and societal trait judgment scales were formulated in an absolute, rather than relative, manner. Finally, to improve internal consistency of the IATs, the number of critical trials was doubled from 20 to 40 per combined block.

The hair attitude IAT (*r* = 0.65) and the race attitude IAT (*r* = 0.64) showed adequate split-half reliability, with higher values than those observed in Study 3, presumably thanks to the larger number of critical trials implemented. The self trait judgment scale (Cronbach’s α = 0.85), the societal trait judgment scale (Cronbach’s α = 0.86), and the legal judgment scale (Cronbach’s α = 0.87) exhibited good-to-excellent internal consistency. The correlation matrix forming the basis of the SEM analysis is reported in Supplementary Table 5 (Supplement 1).

### Results

#### Mean levels

The results involving mean levels of all variables were in line with the results obtained in Studies 1–3, with one notable exception. Specifically, in Studies 2–3, White participants expressed neutrality on the self trait judgment scale, whereas in Study 4, they expressed significant positivity toward Afrocentric hair, *t*(964) = − 16.61, *p* < 0.001, BF_10_ = 1.23 × 10^51^, Cohen’s *d* = − 0.53.

#### Prediction of legal judgments

Similar to Study 3, a latent legal judgment variable was predicted by a latent self trait judgment variable, a latent societal trait judgment variable, a latent implicit hair attitude variable, and a latent implicit race attitude variable. Despite a significant difference between the model-implied and empirical correlation matrices, *χ*^*2*^(109) = 717.29, *p* < 0.001, all alternative measures of model fit indicated excellent fit (see Supplementary Table 3). Therefore, we proceed with model interpretation.^7^

**Fig. 3:**
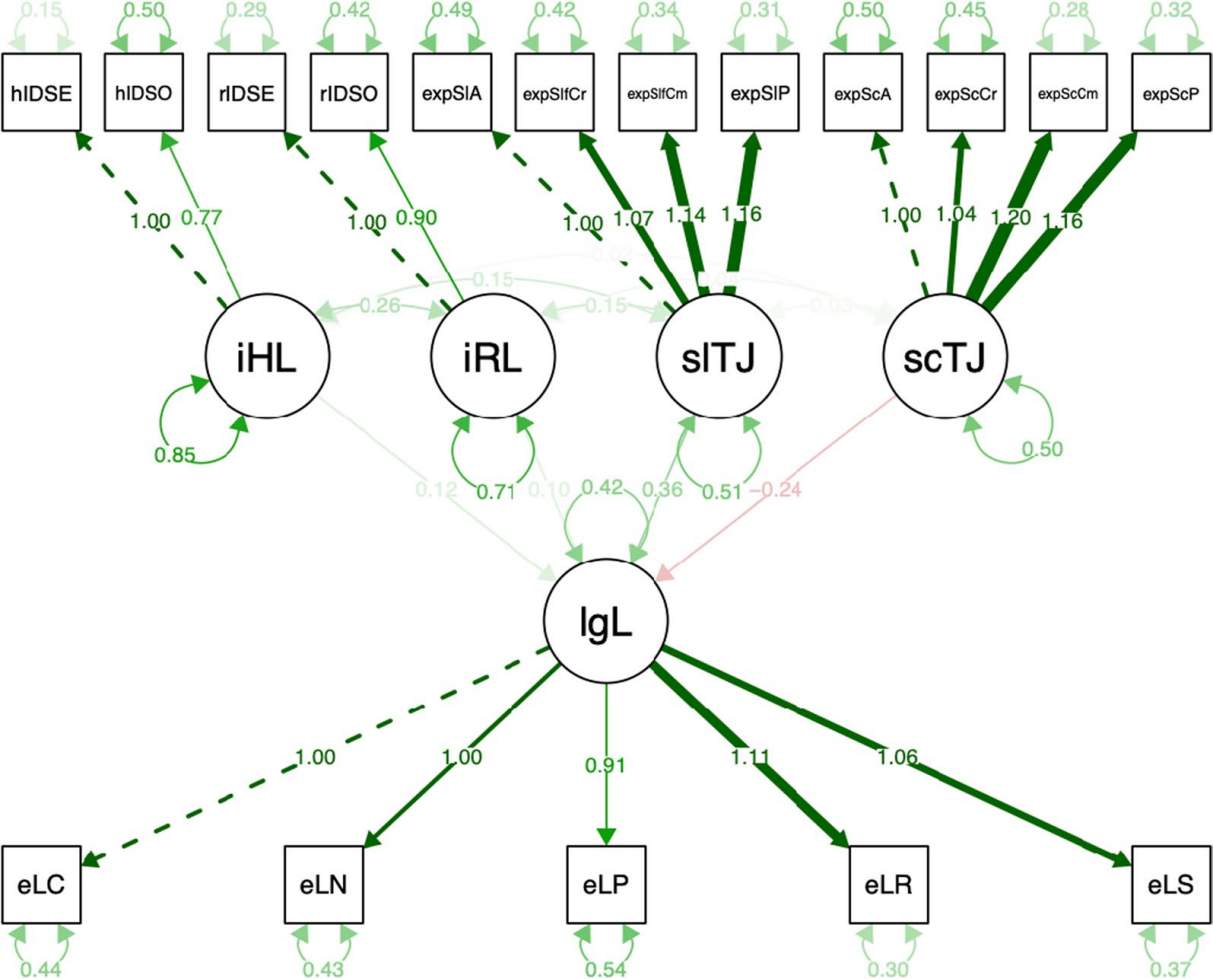
Structural equation model (Study 4) in which a latent legal judgment variable [lgL] is predicted by a latent implicit hair attitude variable [iHL], a latent implicit race attitude variable [iRL], a latent self trait judgment variable [slTJ], and a latent societal trait judgment variable [scTJ]

The results of the SEM model (Fig. 3) replicated the findings of Study 3, with one exception. Specifically, legal judgments were found to be uniquely and positively predicted by self trait judgments, *β* = 0.37 [0.29; 0.45], *z* = 8.94, *p* < 0.001, and uniquely and negatively predicted by societal trait judgments, *β* = − 0.24 [− 0.31; − 0.16], *z* = − 6.37, *p* < 0.001. That is, the higher the level of self-reported preference for Eurocentric over Afrocentric hair texture and the lower the level of acknowledgement of societal bias, the more likely participants were to agree with the corporation rather than with the plaintiff.

Remarkably, in spite of no shared method variance, implicit hair attitudes were again found to be significantly and uniquely associated with legal judgments, *β* = 0.12 [0.06; 0.18], *z* = 3.69, *p* < 0.001. Moreover, unlike in Study 3, the effect of implicit race attitudes on legal judgments was also statistically significant and similar in size, *β* = 0.10 [0.03; 0.17], *z* = 2.81, *p* = 0.005, thus providing further evidence for the idea that the two IATs measure related but separate constructs. At the same time, these results also suggest that the correspondence principle may not apply to implicit social cognition in a straightforward manner. We return to this issue in the General discussion below.

### Discussion

In Study 4, we conducted a direct replication of Study 3 with the safeguards of a power analysis and formal preregistration, along with additional methodological improvements of the IATs and explicit scales. Remarkably, despite these changes, Study 4 confirmed all main findings that had emerged from Study 3 and previous studies: Specifically, White American participants exhibited a significant implicit preference for Eurocentric over Afrocentric hair texture and such implicit preferences predicted expressions of support in a legal case involving discrimination on the basis of hair texture above and beyond the effects of two explicit scales and a White/Black race IAT. As such, this study provides confirmatory evidence for the existence of an implicit hair texture bias among White Americans as well as for the idea that implicit hair texture bias and implicit race bias are related but separate constructs.

In addition, Study 4 also produced two unexpected secondary findings. First, White American participants expressed a significant preference for Afrocentric hair textures on an explicit scale. This finding is in contrast with the results of Studies 2–3 in which participants remained neutral on a similar measure. We can only speculate as to the reasons for this discrepancy across studies.

It is conceivable that participants were more reluctant to express negative evaluations on an absolute (e.g., “I believe that dreadlocks and afros make women look unprofessional”) rather than a relative scale (e.g., “I believe that dreadlocks and afros make women look less professional”). In addition, Studies 1–3 were conducted prior to the national reckoning on race that began in the summer of 2020, whereas data for Study 4 were collected in June 2021. Explicit expressions of positive regard for Afrocentric hair texture may reflect the vast change in at least such verbal statements supportive of Black American attributes. In addition, research has more generally documented decreases in the expression of explicit prejudice on the Project Implicit website over the past decade (Charlesworth & Banaji, 2019). For example, both race and skin tone bias have decreased by 25% toward neutrality over the past 15 years. As such, the movement in a direction of explicit preference for Afrocentric hair textures may reflect a genuine shift in White American participants’ values.

Second, unlike in Study 3 and in Supplementary Study 2, implicit race attitudes were predictive of expressions of support in the legal case above and beyond the effects of the two explicit scales and implicit hair attitudes. Given these contradictory findings, below we report a meta-analysis in which we investigate whether, pooling the data across relevant studies, implicit race attitudes are significantly predictive of the criterion behavior. Moreover, we also probe whether, in line with the correspondence principle (Ajzen & Fishbein, 1977), the hair attitude IAT is generally more predictive of views about the legal case than the race attitude IAT.

## Meta-analysis of incremental predictive validity

Given the mixed findings obtained in Studies 2–4 and Supplementary Study 2 regarding the incremental predictive validity of the race attitude IAT above and beyond the two explicit scales and the hair attitude IAT and some uncertainty over the relative size of the effects associated with the race and hair attitude IATs, we conducted four internal meta-analyses. Model 1 was an intercept-only model in which we estimated the predictive validity of the two IATs in terms of standardized SEM coefficients above and beyond both explicit measures but without entering the other IAT as a predictor. In model 2, we included an indicator variable in the model to test whether the effect sizes associated with the hair attitude IAT and the race attitude IAT differed from each other. Models 3 and 4 were the same as models 1 and 2, with the exception that the effect sizes measured incremental predictive validity of the two IATs not only with regard to the explicit scales but also with regard to each other.

Overall, the results of the four models are straightforward. The two IATs showed incremental predictive validity both above and beyond explicit measures (model 1), *β* = 0.12 [0.09; 0.15], *z* = 7.18, *p* < 0.001, and above and beyond explicit measures and each other (model 3), *β* = 0.10 [0.06; 0.13], *z* = 5.16, *p* < 0.001. However, the size of the effect did not differ across the two IATs in either model 2, *β* = − 0.02 [− 0.08; 0.05], *z* = − 0.51, *p* = 0.612, or model 4, *β* = − 0.02 [− 0.09; 0.05], *z* = − 0.51, *p* = 0.606. Taken together, these results suggest that the hair attitude IAT and the race attitude IAT measure independent constructs. At the same time, under the correspondence principle, it may have been expected that the more specific phenotypicality IAT would be more predictive of responses to a legal case involving that phenotype than a generic race IAT. These data are not in line with this prediction, which makes it questionable whether the correspondence principle can be straightforwardly applied to implicit attitudes, at least under the present conditions.

## Results from African American Participants (Studies 1–4)

The number of African American participants in Studies 1–4 individually was relatively small and, as such, we were unable to analyze their data separately for each study. However, combining the data across all studies made it possible to examine their performance (combined *n* = 520). The results from African American participants were analyzed using Bayesian meta-analyses, with results from White American participants presented for comparison (see Table 5).

**Table 5:** Results of Bayesian meta-analyses probing the mean levels of implicit hair attitudes, self trait judgments, societal trait judgments, and legal judgments among African American and White American participants (Studies 1–4)

	African American participants		White American participants	
	Cohen’s *d*	BF_10_	Cohen’s *d*	BF_10_
Black hair attitude IAT	0.22 [0.12; 0.31]	7.72 × 10^3^	0.68 [0.64; 0.73]	6.42 × 10^191^
Self trait judgments	− 0.63 [− 0.73; − 0.55]	2.32 × 10^31^	− 0.31 [− 0.33; − 0.28]	2.30 × 10^39^
Societal trait judgments	1.42 [1.34; 1.56]	1.80 × 10^110^	1.09 [1.01; 1.13]	5.33 × 10^344^
Legal judgments	− 1.85 [− 1.98; − 1.73]	1.85 × 10^149^	− 1.15 [− 1.21; − 1.11]	4.75 × 10^377^

Cohen’s *d* effect sizes are scored such that more positive scores indicate higher levels of bias in favor of Eurocentric hair texture. 95-percent HDIs (highest density intervals) are reported in square brackets. BF_10_ refers to Bayes Factors providing a relative measure of evidence in favor of the alternative hypothesis (μ ≠ 0) over the null hypothesis (μ = 0)

Similar to White American participants, African American participants exhibited an implicit preference in favor of Eurocentric hair texture; however, this preference was considerably weaker compared to the same bias in White Americans. On explicit self trait judgments, African American participants expressed considerably stronger explicit preference in favor of Afrocentric hair texture than White American participants. Moreover, as reported above, the latter remained neutral on this measure in Studies 2–3 and showed a preference for Afrocentric hair texture only in Study 4. On the societal trait judgment scale, both groups reported similarly high levels of societal bias against Afrocentric hair texture.

On the legal judgment measure, both groups exhibited support for the African American plaintiff, although this effect was markedly larger among African Americans. This result is noteworthy. Even on the most direct measure presented in these studies, which was asking for degree of agreement with a Black plaintiff who simply wanted to wear her hair the way she wished, White and Black Americans were not equal in their support for her over a corporation. Differences in explicit cognition clearly exist between the two groups and are apparent in this comparison.

Finally, perhaps due to a restriction in the range of the legal judgment variable or the relatively small sample size, the SEM analysis of the data obtained from African American participants in Study 3 resulted in poor model fit. As such, we refrain from interpreting the coefficients obtained from this model. In Study 4, model fit indices were satisfactory, but the results diverged markedly from the results obtained in the sample of White American participants reported above. Specifically, scores on the legal judgment scale were predicted exclusively by the societal trait judgment variable, *β* = − 0.21 [− 0.36; − 0.06], *z* = − 2.70, *p* = 0.007. By contrast, the remaining variables, including the self trait judgment scale, *β* = 0.11 [− 0.02; 0.23], *z* = 1.66, *p* = 0.096, the White/Black race attitude IAT, *β* = − 0.00 [− 0.15; 0.14], *z* = − 0.05, *p* = 0.959, and the Black hair attitude IAT, *β* = 0.02 [− 0.08; 0.11], *z* = 0.34, *p* = 0.736, were not significantly associated with the criterion measure. Potential reasons for this discrepancy are explored in more detail in the General discussion below.

## General discussion

We conducted an initial investigation of implicit (i.e., automatically revealed) racial phenotypicality bias, focusing specifically on implicit attitudes toward Afrocentric versus Eurocentric hair texture. Across three high-powered studies, the last of which was formally preregistered, we found that although implicit and more commonly used explicit measures of racial phenotypicality bias were positively correlated with each other, this correlation, similar to the relationship between implicit and explicit race attitudes (Nosek et al., 2007), was small in size. Crucially, implicit and explicit phenotypicality bias each uniquely contributed to the prediction of relevant social behavior among White American participants (Studies 2–4). Moreover, the two also differed in mean levels: Whereas White American participants expressed neutrality on explicit measures of attitude (Studies 2–3), implicit measures revealed a large degree of preference in favor of Eurocentric (straight or wavy) over Afrocentric (curly) hair texture.

Strikingly, in Study 4, for which data were collected in June 2021, White American participants even expressed significantly positive views toward Afrocentric (curly) hair texture on an explicit (self-report) measure of attitude. This difference between Studies 2–3, in which self-reports were at neutrality, and Study 4, in which they showed outgroup-favoring tendencies, may have emerged for a number of reasons. Potentially, participants may have been more reluctant to express even neutrality on an absolute scale (with the endpoints labeled “extremely unprofessional” vs. “extremely professional” or “extremely uncaring” vs. “extremely caring”) rather than a relative scale (with the endpoints labeled “much less professional” vs. “much more professional” or “much less caring” vs. “much more caring”).

More importantly, however, data from the Project Implicit website (e.g., Charlesworth & Banaji, 2019) as well as from representative social surveys (e.g., Nosek et al., 2009) suggest that White Americans have become increasingly reluctant to express views that they believe may be seen by others as biased. What counts as biased is, of course, multiply determined, but social norms are known to play a key role in this regard (Tankard & Paluck, 2016). As such, as more jurisdictions follow the 11 states already banning discrimination based on hair texture as of this writing (California, New York, New Jersey, Virginia, Colorado, Washington, Maryland, Connecticut, New Mexico, Delaware, and Nebraska), social norms may shift even more strongly toward making it unacceptable to express negative views of natural hairstyles. If this is, indeed, going to be the case, then we anticipate that the importance of implicit measures, which are less amenable to voluntary control in line with participants’ egalitarian values, will only gain in importance in the study of racial phenotypicality biases in the coming years. Such considerations apply particularly strongly to political liberals, such as the majority of the Project Implicit participants recruited for the present studies, who are known to be especially sensitive to concerns related to equity and fairness (e.g., Graham et al., 2009).

Explicit and implicit phenotypicality bias were also clearly dissociated among African American participants: Whereas the former revealed preference for Afrocentric hair texture, the latter revealed preference for Eurocentric hair texture. This result is reminiscent of other findings among members of historically disadvantaged social groups who often express preference for the ingroup on explicit measures but tend to reveal neutrality or even outgroup-favoring tendencies on implicit measures (Nosek et al., 2007). Such effects may emerge as a result of members of such social groups internalizing prevalent societal attitudes, as a result of personal experiences with discrimination, and a number of other factors. As such, the present project suggests that future studies of racial phenotypicality bias may benefit from the inclusion of implicit measures, such as the Implicit Association Test (Greenwald et al., 1998), in addition to more commonly used self-report measures not only among White American but also among Black American participants.

Notably, more closely investigating the antecedents and correlates of hair texture bias among Black Americans may be especially instructive with regard to understanding the mechanisms underlying such effects, on which the present data are relatively silent. For example, implicit negativity toward natural hair may stem from perceptions of professionalism (e.g., Koval & Rosette, 2020; Opie & Phillips, 2015). Additionally, or alternatively, natural hairstyles may be perceived to be associated with political causes such as the Black liberation movement of the 1960s and its present-day successors, whereas chemically relaxed hairstyles may be perceived to be associated with assimilation into White culture. In this context, it may be particularly instructive to probe how (implicit) self-esteem and (implicit) self-concept are associated with implicit hair attitudes specifically among Black Americans. Notably, predictive validity of the hair attitude IAT among Black Americans may be improved by using more culturally appropriate category labels (e.g., “natural” vs. “relaxed”) instead of “curly” versus “straight”, which were selected primarily with White American participants in mind.

Importantly, in line with existing theoretical perspectives (Maddox, 2004) and empirical findings (Blair et al., 2004), the present data also suggest that implicit phenotypicality bias and implicit race bias, although statistically related to each other, are far from redundant. Specifically, implicit race attitudes accounted for only about 20 percent of the meaningful variance in implicit hair attitudes (Study 1). Moreover, when data were aggregated across studies, both implicit hair attitudes and implicit race attitudes significantly predicted a measure of intergroup behavior, specifically expressions of support toward a Black plaintiff who sued a corporation alleging discrimination on the basis of her natural hairstyle, among White American participants (Studies 2–4). Notably, each IAT did so incrementally, after accounting for the effects of the other IAT and the effects of explicit measures. As such, these data are clearly in conflict with recent perspectives questioning whether implicit and explicit (race) attitudes represent different constructs.

The latter finding regarding predictive validity is both methodologically and theoretically informative. When it comes to the methodological features of the present project, in investigating predictive validity, we sought to implement several recommendations formulated in the recent meta-analysis by Kurdi et al. (2019b). Specifically, the sample size in the present studies was over ten times larger than the median effect size in studies investigating predictive validity of the IAT and related measures. This distinction is made especially relevant by the fact that the mean effect size revealed by the meta-analysis for both implicit (*β* = 0.14) and explicit predictors (*β* = 0.11) was small. In addition, also in line with recommendations by Kurdi et al. (2019b) and more general recommendations by Nosek et al., (2018, 2019), we confirmed the exploratory results emerging from Studies 1–3 in a preregistered and highly powered final study (Study 4). Finally, and crucially, we made sure that the to-be-predicted behavioral measure had excellent validity and internal consistency. As is well known from classical test theory, noisy criterion measures, and especially unvalidated one-shot measures of behavior, impose a (sometimes quite severe) upper limit on the predictive validity of attitude measures (e.g., Furr & Bacharach, 2013). On a related note, we explicitly took measurement error into account by relying on a structural equation modeling approach.

When it comes to the theoretical implications of the present work for the attitude–behavior relationship, based on the correspondence principle (Ajzen & Fishbein, 1977) and recent empirical findings by Irving and Smith (2020), we expected that a specific implicit attitude (toward Afrocentric hair texture) would be more predictive of a specific behavior (views about discrimination based on Afrocentric hair texture) than a more general implicit measure of race attitudes. Although some individual studies included in the present paper seemed to provide initial evidence in favor of this idea, in the more highly powered last study, both implicit hair attitudes and implicit race attitudes were equally associated with the relevant criterion behavior. Moreover, this finding was also confirmed in an internal meta-analysis combining data from all relevant studies.

Do these findings imply that the correspondence principle is irrelevant to implicit social cognition? We believe that this conclusion is premature, for a number of reasons. Notably, we used the correspondence principle in making multiple decisions related to design of the hair attitude IAT. For example, we used only images of women and only images of Black individuals on the basis of the correspondence principle. Specifically, we reasoned that views on a legal case involving discrimination against a Black woman would best be predicted by an IAT that included stimuli only of the relevant race and gender, especially given that “male” and “White” are perceived as default categories in present-day American society (e.g., Bosson et al., 2018). Moreover, we may have failed to find direct evidence for the correspondence principle in the present studies because hair may be a relatively central and prototypical feature of Black women. The incremental predictive validity of the more specific IAT may have been higher for a feature that is less centrally associated with the overarching group representation itself.^8^

We hope that future work will more systematically explore the operation of the correspondence principle in the context of the IAT and implicit social cognition more generally. When it comes to the IAT specifically, the correspondence principle may operate either at the level of attributes and attribute stimuli or at the level of categories and category stimuli. For example, when it comes to attribute stimuli, one may reason that specific attributes (such as “professional” vs. “unprofessional”) may be more predictive of the legal judgments in the present studies than general attributes (such as “good” vs. “bad”) given that the legal case centers specifically on the question of whether natural Black hair and professionalism are compatible with each other. However, in this context, considerable added complexity may be created by the fact that stereotype IATs using clearly valenced attributes are usually highly correlated, or even redundant, with attitude IATs (Kurdi et al., 2019a; Phills et al., 2020).

Alternatively, or in addition, correspondence may be thought of as applying to the category labels or category stimuli used on the IAT. For example, in Studies 1–3 we used a race IAT that had both male and female faces as category stimuli, whereas in Study 4 we used a race IAT that had only female faces as category stimuli. Based on the correspondence principle, it may have been expected that the race IAT in Study 4 would be more predictive of the criterion behavior given that the criterion behavior related specifically to discrimination against a Black woman. However, IAT effects tend to generally be more strongly influenced by category (and attribute) labels than by the specific category (and attribute) stimuli (e.g., Axt et al., 2021; Mitchell et al., 2003). As such, the predictive validity of the race IAT may have been improved had the category labels been changed to “White women” and “Black women” from “White people” and “Black people”. These specific variations, and other variations along similar lines, may be more systematically explored in future work.

Moreover, in Studies 2–3, implicit hair attitudes predicted a behavior (judgments regarding a legal case) that was, to a large extent, under participants’ volitional control. As such, the present project provides evidence in favor of another idea proposed by Kurdi et al. (2019b), namely that, contrary to the assumptions of several dual-process theories, implicit attitudes may be associated not only with relatively automatic but also with relatively controllable behaviors. At the same time, it should be noted that legal judgments were more strongly predicted by explicit judgments than by implicit attitudes. However, to a considerable degree, this effect is likely to have been due to method variance shared between the explicit scale and the to-be-predicted behavior (Campbell & Fiske, 1959). Moreover, third variables, including drive for consistency between attitudes and behavior (Festinger & Carlsmith, 1959) and motivation to control prejudiced responding (Plant & Devine, 1998), may also have played a role.

Given that, to our knowledge, the present paper reports the first peer-reviewed studies demonstrating the existence and downstream consequences of implicit phenotypically bias more generally and implicit bias against Afrocentric hair more specifically, the present studies should be understood as providing an existence proof argument. We hope that research in social cognition will continue to investigate implicit racial phenotypicality biases and their relationship with social behavior and further clarify some issues left unaddressed here.

One such issue concerns stimulus variation. Specifically, although Studies 2–3 and Study 4 yielded converging results despite differences in stimulus materials, future work may further explore other possible variations in the IAT procedure. For example, in the present studies we used only relatively dark-skinned Black targets; in the future, investigators may want to ask whether the effect extends to light-skinned Black targets. Similarly, in the present studies we used only female targets; future work may want to examine generalization to targets of other genders. Moreover, the mean level and predictive validity estimates provided by IATs relying on real facial images may be different from the estimates provided by the IATs in the present studies, which all relied on line drawings. Finally, although the present data suggest that hair texture bias emerges especially strongly in the context of Black (female) targets, future work may want to more systematically explore variation by race both in terms of mean levels and in terms of predictive validity.

Given that the studies reported above rely exclusively on the Implicit Association Test, it is unclear to what extent implicit attitudes toward Eurocentric versus Afrocentric hair texture are automatically activated without specific instruction to categorize stimuli along this dimension. Future work using alternative implicit measures of attitudes, such as the evaluative priming task (Fazio et al., 1995) or the affect misattribution procedure (Payne et al., 2005), may be used to investigate this issue. We view such replications and extensions of the present work as especially important given that performance on different implicit measures of attitude has been suggested to emerge from overlapping but not fully identical cognitive processes (De Houwer & Moors, 2010). Similarly, based on the present data, it is unclear whether and to what extent implicit phenotypicality bias would predict criterion behaviors other than the one chosen for the present studies. Specifically, it may be of interest to move away from tightly controlled but relatively artificial settings toward exploring more ecologically realistic behaviors. However, we note that in doing so, it is important to keep in mind considerations regarding the validity and internal consistency of the to-be-predicted behaviors (e.g., Kurdi et al., 2019b).

Finally, recent work in implicit social cognition has demonstrated striking similarities between response time-based measures of implicit attitudes administered to individual participants and algorithmically derived measures of semantic association obtained from large corpora of online text (e.g., Caliskan et al., 2017; Kurdi et al., 2019a; for a review, see Caliskan & Lewis, 2020) and online images (e.g., Steed & Caliskan, 2021). Moreover, relative to the usually modest relationship found between implicit (and explicit) attitudes and intergroup behavior at the level of individuals, studies investigating the same relationship at the level of geographic units tend to reveal considerably larger effects (Hehman et al., 2019; Payne et al., 2017). We hope that future work will be able to combine these novel approaches with the idea suggested by the present project, namely that focusing on specific features of the attitude object (e.g., biases based on specific phenotypic traits) may improve the prediction of intergroup behavior, to investigate the precursors, magnitude, and downstream consequences of explicit and implicit social evaluations beyond the laboratory.

## Data Availability

All data files, analysis scripts, and stimuli used in this project are available for download from the Open Science Framework: https://osf.io/xn7az/.

## Footnotes

1 In social psychology, the term “behavior” is often used to exclusively refer to ecologically valid expressions of behavior, such as smiling at someone, donating money to an organization, or participating in a religious ritual. In this article, we follow the traditions of experimental psychology in using “behavior” in a more general sense, to distinguish a class of measures from direct measures of brain activation or physiology (e.g., Niv, 2021). Under this definition, the distinction between attitudes and behaviors becomes meaningless given that performance on explicit or implicit measures of attitude can itself be seen as a specific type of behavior (e.g., De Houwer, 2019).

2 We considered multiple alternatives to refer to the two hair textures whose effects are investigated in the present work, including “Eurotextured” versus “Afrotextured,” “curly” versus “straight,” and “natural” versus “relaxed.” However, to highlight connections of the present work with the broader research tradition on racial phenotypicality bias, we ultimately decided to use “Eurocentric” versus “Afrocentric.” Needless to say, as with any other feature labeled “Eurocentric” versus “Afrocentric,” we do not mean to suggest that White or Black Americans do not exhibit variability on these features, nor do we intend to rarefy or essentialize those differences. However, we recognize that, presumably given statistical regularities, such features come to be associated with racial categories in perceivers’ minds.

3 Data from other groups of participants (such as Asian and Latinx participants) are available for analyses in the open data.

4 A multiple regression analysis of the same data yielding similar conclusions is reported in Supplement 1.

5 In line with the finding reported above that implicit hair attitudes and implicit race attitudes are distinct (Study 1A), an alternative model in which the implicit hair and implicit race attitude indicators loaded on the same latent implicit attitude variable resulted in poor model fit and was thus discarded (for details, see Supplementary Table 3).

6 The less stringent multiple regression analysis of the same data yielded significant and positive coefficients for both IATs (see Supplement 1), thus highlighting the importance of taking into account measurement error when probing incremental predictive validity (Kurdi et al., 2019b; Westfall & Yarkoni, 2016). In Supplementary Study 2 reported in Supplement 1, we obtained a similar result in a model that included only a White/Black race attitude IAT but no hair attitude IAT. In this study, legal judgments were significantly predicted by self and societal trait judgments irrespective of the method of analysis chosen. However, implicit White/Black race attitudes were found to be predictive only in a multiple regression, but not an SEM, analysis.

7 In line with the finding reported above that implicit hair attitudes and implicit race attitudes are distinct (Studies 1A and 3), an alternative model in which the implicit hair and implicit race attitude indicators loaded on the same latent implicit attitude variable resulted in poor model fit and was thus discarded (for details, see Supplementary Table 3).

8 We thank an anonymous reviewer for raising this idea.
